# Oxidative Stress-Induced Alteration of Plant Central Metabolism

**DOI:** 10.3390/life11040304

**Published:** 2021-04-01

**Authors:** Tatyana Savchenko, Konstantin Tikhonov

**Affiliations:** Institute of Basic Biological Problems, Pushchino Scientific Center for Biological Research, Russian Academy of Sciences, 142290 Pushchino, Russia; ktikhonov@rambler.ru

**Keywords:** oxidative stress, central metabolites, sugars, amino acids, tricarboxylic acid cycle metabolites

## Abstract

Oxidative stress is an integral component of various stress conditions in plants, and this fact largely determines the substantial overlap in physiological and molecular responses to biotic and abiotic environmental challenges. In this review, we discuss the alterations in central metabolism occurring in plants experiencing oxidative stress. To focus on the changes in metabolite profile associated with oxidative stress per se, we primarily analyzed the information generated in the studies based on the exogenous application of agents, inducing oxidative stress, and the analysis of mutants displaying altered oxidative stress response. Despite of the significant variation in oxidative stress responses among different plant species and tissues, the dynamic and transient character of stress-induced changes in metabolites, and the strong dependence of metabolic responses on the intensity of stress, specific characteristic changes in sugars, sugar derivatives, tricarboxylic acid cycle metabolites, and amino acids, associated with adaptation to oxidative stress have been detected. The presented analysis of the available data demonstrates the oxidative stress-induced redistribution of metabolic fluxes targeted at the enhancement of plant stress tolerance through the prevention of ROS accumulation, maintenance of the biosynthesis of indispensable metabolites, and production of protective compounds. This analysis provides a theoretical basis for the selection/generation of plants with improved tolerance to oxidative stress and the development of metabolic markers applicable in research and routine agricultural practice.

## 1. Introduction

Throughout their life, plants often experience oxidative stress, which occurs due to the shift in the dynamic balance between the rates of formation and removal of reactive oxygen species (ROS) [1]. At moderate concentrations, ROS serve as an important component of the signal transduction system [2]. There are a number of protective processes that require significantly increased levels of ROS for their implementation, for example an increased amount of ROS is needed to build up a protection against pathogens by inducing the cross-linking of cell wall polymers or initiating programmed cell death. However, an uncontrolled increase in ROS levels leads to the accumulation of oxidative damage of cellular components [3]. In plant cells, ROS formation occurs in the chloroplasts, peroxisomes, mitochondria, plasma membrane, endoplasmic reticulum, and cell wall, however, growing evidence suggests that chloroplasts are the major contributors to oxidative stress [4].

Oxidative stress is an integral component of various stress conditions, including excessive light, cold, heat, drought, wounding, waterlogging, microbial diseases, and atmospheric and soil pollutants, and this fact largely determines the substantial overlap in plant physiological and molecular responses to various environmental challenges, biotic and abiotic [5,6,7]. Nevertheless, there are several experimental approaches, which allow the dissection of the traits associated with oxidative stress per se: (1) exogenous application of hydrogen peroxide or redox agents, such as cycling quinones menadione and paraquat (methyl viologen) [6,8,9,10]; (2) plant treatment with hormones inducing oxidative stress responses, such as jasmonic acid, abscisic acid, and salicylic acid [11,12,13]; and (3) analysis of mutants displaying a constitutive/altered oxidative stress response [14,15,16,17,18].

It has been established that under various stress conditions the presence of a “oxidative constituent” manifests itself through the alteration of physiological parameters (growth inhibition, chlorophyll bleaching, hypersensitive response-like cell death) and biochemical characteristics (changes in enzymatic and nonenzymatic antioxidants levels; damage to membranes, DNA, RNA, and protein molecules; accumulation of lipid peroxidation products, etc.). The distinct metabolic changes, which are repeatedly observed under different stress conditions and display correlation with enhanced ROS production, accumulation of lipid peroxidation products, and induction of the antioxidant system, can also be considered as symptoms of oxidative stress [6,19,20,21]. Numerous studies have described oxidative stress-induced alterations in the content of small molecules, including ROS themselves, antioxidants, and redox compounds (reviewed in [22]). At the same time, abundant evidence points to the specific changes in amino acids, major sugars, and their derivatives occurring under oxidative stress conditions [9,14,17,23,24,25]. Adjustments of central metabolic fluxes under oxidative stress conditions allow plants to prevent the accumulation of ROS, maintain the biosynthesis of indispensable metabolites, and initiate the production of protective compounds. Reliable and specific metabolic biomarkers of oxidative stress are already routinely used to diagnose pathophysiological conditions and diseases in medical research [26,27]. In plants, the identification of characteristic metabolic markers of oxidative stress is associated with difficulties related to the dynamic and transient character of stress-induced changes in metabolites, a strong dependence of metabolic responses on the intensity of stress, duration of exposure to stress conditions, and a significant variation in responses among different plant species and tissues [28,29]. Thus, further comparative analysis and systematization of relevant accumulated metabolomics data are still needed to develop a fundamental base for a better understanding of the mechanisms of plant tolerance to oxidative stress and the development of metabolic markers applicable in research and routine agricultural practice.

## 2. Reactive Oxygen Species Types and Origin in Plant Cell

ROS are formed from O_2_ in electron transport chains, in non-enzymatic chemical reactions, in several enzymatic reactions, and during photosensitization, the reaction of O_2_ with a photoexcited molecule [1,4,30]. The major ROS are singlet oxygen ^1^O_2_, superoxide anion radical O_2_^−^ hydrogen peroxide H_2_O_2_, and hydroxyl radical OH. Singlet oxygen ^1^O_2_ is produced through the interaction of triplet oxygen ^3^O_2_ with a chlorophyll molecule in the triplet state [31], mainly at the photosystem II (PSII) reaction center when the electron flux from PSII is hampered [30,32]. Alternatively, O_2_ can undergo a one-electron reduction in electron transport chains to form the superoxide anion radical O_2_^−^. The main place of O_2_^−^ formation is the acceptor side of PSI (Mehler reaction) [33,34,35]. O_2_^−^ is eliminated through a disproportionation reaction leading to the formation of H_2_O_2_ and O_2_, occurring spontaneously or carried out enzymatically with the participation of superoxide dismutase [36]. H_2_O_2_ is also produced in large amount in peroxisomes during photorespiration [37], the initial steps of which take place in the chloroplasts. In the chloroplast, the ascorbate peroxidase system and peroxiredoxins play an important role in the elimination of H_2_O_2_ [3,35,38]. The one-electron reduction of the H_2_O_2_ molecule catalyzed by free ions of Fe^2+^ and other transition metals (Cu^+^, Mn^2+^) results in the formation of a hydroxyl radical OH (Fenton reaction) [39]. Hydroxyl radical OH is the most dangerous ROS since it reacts with all biological molecules right at the site of origin forming organic radicals and triggering radical chain reactions [30]. The oxidative stress within the cell is mainly transmitted by H_2_O_2_ molecules, which are less reactive and relatively long living [4].

The most common cause of oxidative stress in plants is the saturation of the photosynthetic electron transport chain, which leads to the increased production of O_2_^−^ and ^1^O_2_. This saturation is promoted by excessive illumination, and also a reduced rate of CO_2_ fixation [4]. CO_2_ deficiency can occur as a result of rapid assimilation under high light conditions or due to the stomata closure caused by drought and high temperatures. In addition to over-reduction of the photosynthetic electron transport chain, the lack of CO_2_ leads to an increase in photorespiration and the production of H_2_O_2_ in peroxisomes [37]. The low temperatures lead to a significant reduction in the activity of the enzymes of the Calvin cycle and antioxidant enzymes, while light absorption processes are suppressed only slightly. This disbalance causes oxidative stress [1]. Heavy metal ions have a high affinity for protein cysteine residues. Therefore, heavy metal poisoning leads to the inactivation of antioxidant enzymes and enzymes of the Calvin cycle [40]. Moreover, transition metal ions catalyze the Fenton reaction and thus increase the production of hydroxyl radicals. Oxidative stress can be induced by herbicides, such as methyl viologen, transferring electrons from the photosynthetic chain to O_2_, or diuron and atrazine, blocking the electron transport chain and increasing the production of O_2_^−^ [1].

Thus, it is clear that in plant cells, the processes occurring within chloroplasts are the major contributors to oxidative stress. At present, the attention of researchers has largely shifted to the study of the fundamental signaling functions of ROS and their interaction with other signaling systems [2,41,42,43]. Importantly, ROS transfer signals, not only within the cell, but also between different plant organs [43,44,45]. The damaging and the signaling activities of ROS will lead to different effects on the level of gene expression profile and metabolism, including the activation of the mechanisms that are simply targeted at the removal of ROS (i.e., the induction of the antioxidant system), or the induction of mechanisms with broader adaptive consequences accompanied by the transcriptomic reprogramming and redistribution of metabolic fluxes. Interestingly enough, these effects can manifest themselves independently of one another. For example, in rice, the overexpression of glycolate oxidase confers tolerance to high light and increased temperature through the involvement of H_2_O_2_ as a signaling molecule triggering the protective responses, and does not activate the antioxidant protection [46].

## 3. Metabolic Signature of Oxidative Stress in Plants

Control over adaptation to stress conditions is implemented through the activity of a multilevel regulatory system, which includes mechanisms controlling gene expression on transcriptional and post-transcriptional levels and secondary protein modifications [47]. The adaptation to oxidative stress is accompanied by the active regulatory events at the post-transcriptional level and metabolism level [6,24,48], and because of this, the analysis of alteration of gene expression at a transcription level does not allow fully understanding the essence of the ongoing adaptive processes. Besides this, the enzyme capacity in the central metabolism is largely in excess, and the metabolic fluxes to a great extent are regulated via simple mass action across the metabolic network [49]. Thus, the assessment of the levels of selected metabolites may provide reliable information about a plant’s physiological status and allow the identification of the processes primarily affected by oxidative stress.

### 3.1. Sugars and Sugar Derivatives

**Figure 1:**
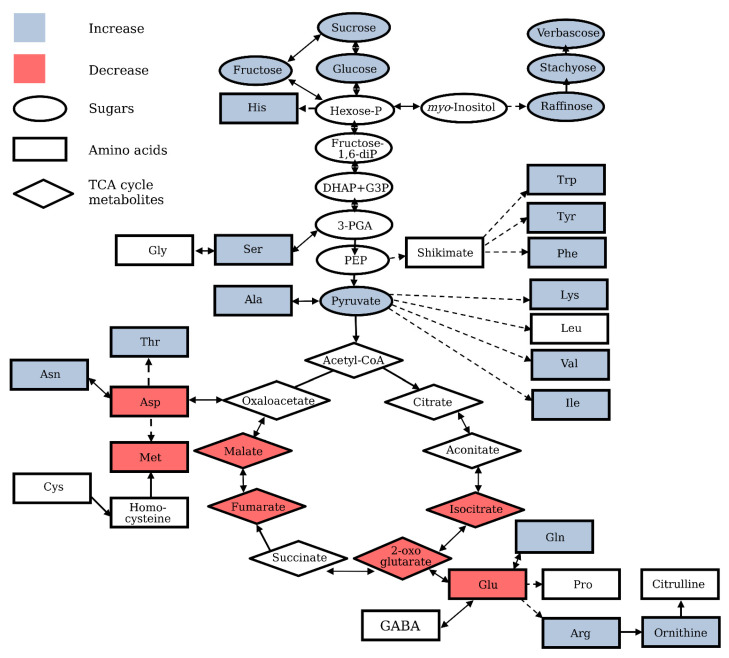
Oxidative stress-specific alteration of central metabolism in plants. Schematic representation of the major metabolic pathways and central metabolites, sugars, amino acids, and tricarboxylic acid (TCA) cycle metabolites. Based on the results of multiple studies, the levels of numerous metabolites were shown to be affected by oxidative stress, increased (indicated in blue) or decreased (indicated in red). Please note, that the scheme reflects the trends observed in the majority of studies, although the individual metabolites may display a different pattern in selected studies.

Major rearrangements of metabolic pathways under oxidative stress conditions observed in independent studies include the down-regulation of the glycolysis and tricarboxylic acid (TCA) cycle and activation of the oxidative pentose phosphate pathway [9,14,17,24,28]. The rerouting of glycolytic carbon flow into the oxidative pentose phosphate pathway mainly determines the changes in the sugar profile and alters the levels of sugar phosphates and soluble carbohydrates with antioxidant properties (Figure 1) [9,25]. Antioxidant properties have been confirmed for numerous soluble carbohydrates, such as sucrose, fructose, raffinose, sorbitol, mannitol, and fructans in plant tissues, in vitro, and in specific model systems, such as fish oil-in-water emulsion [21,50,51,52,53,54,55,56,57]. Being directly associated with ROS-producing processes, such as respiration and photosynthesis, and simultaneously with anti-oxidative processes, such as the oxidative pentose-phosphate pathway and carotenoid biosynthesis, soluble sugars (mainly mono- and disaccharides) are involved in the formation of the prooxidant and antioxidant intracellular environment [50,58]. Based on the strong antioxidant properties of sucrose, a primary stable carbohydrate of photosynthesis and major transported sugar in plants, the antioxidant functions of this metabolite in plant tissue have been suggested, especially in tissues with a high sucrose content [59,60,61]. Fast accumulation of sucrose and fructose, preceded by an increase in glucose level, was observed in the *Arabidopsis* cell culture treated with H_2_O_2_ [24]. High ozone concentrations known to induce oxidative stress in plants [62] led to a significant increase in water soluble carbohydrates in leaves of tree species *Phoebe bournei* and *Phoebe zhennan* [63]. Transgenic potato plants with increased sugar content resulted from the insertion of a yeast-derived invertase gene, coding for sucrose metabolism enzyme, and displayed a higher tolerance to hypothermia-induced oxidative stress, wherein the protective effect of sugars was caused by their ability to scavenge ROS nonspecifically under stress conditions [64]. The absence of either alkaline or neutral invertases in *Arabidopsis* was associated with higher expression of the oxidative stress defense gene, while transient overexpression of invertase gene in leaf mesophyll protoplasts downregulated the oxidative stress-responsive promoter of ascorbate peroxidase 2 [65]. The reprogramming of sugar metabolism under oxidative stress conditions was also confirmed by the decreased starch accumulation in transgenic *Arabidopsis* plants constitutively experiencing oxidative stress due to the overexpression of glycolate oxidase gene [18]. It is important to keep in mind that sugars perform not only antioxidant, but also signaling, functions. This, in particular, is confirmed by the data revealing the requirement of mitochondria-associated hexokinase for the development of oxidative stress-induced programmed cell death (PCD) in plant cells, suggesting a link between the signaling functions of glucose and apoptosis [66].

Stresses that give rise to excess concentrations of ROS, such as drought, freezing, heat, and excessive light irradiation, are accompanied by an accumulation of raffinose family oligosaccharides (RFO), which are α-galactosyl extensions of sucrose [67,68,69]. Among these oligosaccharides, raffinose was found in many plants, and oligosaccharides of a higher degree of polymerization, such as stachyose and verbascose, were present only in selected species [70], where they function as osmoprotectants and stabilizers of cellular membranes. Several lines of evidence suggest that these metabolites are involved in plant adaptation to conditions of oxidative stress [59,60,68] and act as antioxidants and scavengers of hydroxyl radicals, the most deleterious form of ROS [67]. The induction of genes of galactinol synthase and raffinose synthase, the enzymes of the RFO biosynthesis pathway, and heat shock transcription factors regulating the expression of these genes by hydrogen peroxide treatment, confirms the specific connection between this particular pathway and oxidative stress [68]. It has been established that raffinose accumulation in *Arabidopsis* is not regulated by the abscisic acid (ABA)-dependent CBF/DREB1 (the C-repeat (CRT)-binding factor/dehydration-responsive element (DRE) binding protein 1) pathway [71], but *Boea hygrometrica* genes of galactinol synthase and raffinose synthase contain W box *cis*-elements in their promoters, known targets for the ABA-inducible WRKY family of transcription factors that mediate oxidative stress responses [72,73]. The overexpression of galactinol synthase, raffinose synthase, or the heat shock transcription factor A2, leading to an increase in intracellular levels of RFO in transgenic *Arabidopsis* plants, resulted in the improvement in ROS scavenging capacity and oxidative stress tolerance [68,74,75]. The presence of specific raffinose transporters, enabling the transport of RFO to chloroplasts, justifies the antioxidant functions of these metabolites in plastids [76]. The level of *myo*-inositol, a substrate for galactinol biosynthesis [77,78], which is in turn a substrate for raffinose biosynthesis, also was reported to be altered under oxidative stress conditions [21,79,80]. In apple, *myo*-inositol regulates ROS-induced programmed cell death through the salicylic acid-dependent and ethylene-dependent pathways [79]. *Arabidopsis* mutant for a specific *myo*-inositol phosphate synthase (MIPS1) under long day conditions or at increased growth irradiance shows a lesion phenotype that is similar to the hypersensitive response used by plants to prevent the spreading of infection [81,82]. Since *myo*-inositol also serves as a substrate for the biosynthesis of other metabolites and signaling molecules, contributing to plant adaptation to adverse environmental conditions, the level of this metabolite does not necessarily increase under stress conditions [83]. For instance, in *Arabidopsis cat2* mutant, which is constitutively in a state of oxidative stress due to the absence of the functional *CATALASE2* gene, *myo*-inositol level was reduced under stress conditions [15,84].

Mannose, an epimer of glucose, is directly involved in the biosynthesis of the major antioxidant, ascorbate [85], and mannose derivative mannitol has protective functions under oxidative stress conditions. Transgenic *Nicotiana tabacum* plants, overexpressing mannitol-1-phosphate dehydrogenase gene coding for the chloroplasts protein, and, consequently, accumulating an increased level of mannitol in chloroplasts, display enhanced tolerance to methyl viologen-induced oxidative stress [55]. Threonate, a derivative of four-carbon monosaccharide threose, was also observed to accumulate in menadione-treated *Arabidopsis* cells [9], though it is important to remember that threonic acid can be formed from threose or as a result of the degradation of ascorbate [86].

Among the known di- and oligosaccharides with protective functions under oxidative stress conditions, the most studied are trehalose, a disaccharide consisting of two glucose molecules, and short-chain fructans, polymers of fructose molecules terminated by a single glucose molecule. Foliar application of trehalose led to a significant decrease in symptoms of oxidative stress in quinoa and wheat plants [87,88]. Transgenic tomato plants, accumulating higher levels of trehalose due to the overexpression of yeast trehalose-6-phosphate synthase gene, exhibited enhanced tolerance to oxidative stress [89]. The antioxidant properties of fructans have been demonstrated in plants and intestinal lumen [54,90,91]. Furthermore, in plants the metabolism of fructans is modulated by foliar application of S-nitrosoglutathione, a NO donor, wherein an increase in the fructans level helps to mitigate oxidative stress [91]. Importantly, fructans also scavenge hydroxyl radicals, and a predominant outcome of the ·OH–fructans interaction is the splitting of oligosaccharides, resulting in the formation of non-radical products, which can be used as markers for nonenzymatic sugar–radical interactions in vivo [54].

While all the above-mentioned sugars were shown to be involved in the oxidative stress response, a certain specificity of their protective functions is evident. For example, tolerant rice species accumulated galactose and raffinose under chill-stress conditions whereas these saccharides declined in sensitive species, and at the same time, the tolerance mechanism in the more salt- and water-deficit-tolerant species was associated with the accumulation of osmoprotectants, such as glucose, trehalose, and mannitol [92].

### 3.2. Tricarboxylic Acid Cycle Metabolites

Many tricarboxylic acid (TCA) cycle intermediates serve as substrates for several biosynthetic pathways, and therefore their levels display a very dynamic character, reflecting the processes of formation and consumption of these metabolites. Nevertheless, the levels of TCA cycle metabolites have been shown to increase when respiration is stimulated [93], and the characteristic decrease in the pool of TCA cycle metabolites has been observed in plant cells under oxidative stress conditions (Figure 1) [6,10,14]. Modulation of the activity of TCA cycle enzymes seems to contribute significantly to the alteration of the metabolic profile under oxidative stress conditions. Many enzymes of the tricarboxylic acid cycle, such as aconitase, pyruvate-dehydrogenase, and 2-oxoglutarate-dehydrogenase, are sensitive to oxidative inhibition [25,94,95,96], while other enzymes have been shown to be subject to redox modifications under stress conditions (reviewed in [10]). A decrease in TCA cycle metabolites was detected in heterotrophic *Arabidopsis* and rice cultured cells [9,14,24] and roots under oxidative stress conditions [25,28]. The suppression of operation of mitochondria TCA cycle activity in nonphotosynthetic cells upon menadione treatment [9,14,25,28] is rather expected, because the mitochondrion is one of the main sites of ROS production upon addition of menadione to nonphotosynthetic cells. H_2_O_2_ and methyl viologen treatments also affect the TCA cycle significantly [17,24]. Unfortunately, it is impossible to compare the effects induced by menadione, methyl viologen, and H_2_O_2_, since, to best of our knowledge, a comparative analysis of the effects of different treatments on the metabolic profile of the same biological sample has not been carried out yet.

A kinetic analysis of ^13^C-labeled compounds in *Arabidopsis* cultured cells and roots, following labeling with exogenously supplied ^13^C-glucose, confirmed a reduction of carbon flux through the TCA cycle, manifested in decreased labeling of almost all metabolites under menadione-induced oxidative stress conditions [9,28]. Expectedly, such a reduction in the carbon flow through the TCA cycle was accompanied by a steady accumulation of pyruvate, which upon oxidation produces acetyl-CoA, feeding the TCA cycle. Interestingly, a decrease of carbon flux through TCA cycle intermediates is not necessarily accompanied by a decreased metabolite abundance, due to the simultaneous decrease in the activity of downstream pathways; and the pattern of changes in the amount of individual TCA metabolites can be dynamic and variable [28]. For example, the citrate abundance was significantly decreased after 0.5 h of treatment with menadione, but then increased steadily under stress conditions, and even during the recovery from stress, the 2-oxoglutarate level showed a transient increase after the menadione treatment, then decreased below control level in 2 h, followed by three-fold control level increase in 6 h after the treatment. Succinate, fumarate, and malate were decreased up to two-fold [28]. A significant decline in the levels of glycolytic and TCA cycle intermediates was also observed in wild type *Arabidopsis* upon methyl viologen treatment, reflecting oxidative stress in plastids and inhibition of photosynthesis and respiration [17]. The pyruvate and TCA cycle metabolites displayed the most prominent differences between methyl viologen-treated wild type *Arabidopsis* and *rcd1* (*radical-induced cell death1*) mutant, showing improved tolerance to methyl viologen-induced oxidative stress [17]. In *rcd1* mutant, the levels of all TCA cycle intermediates, except fumarate, increased or remained high after methyl viologen treatment.

Although most studies have pointed to the suppression of TCA cycle activity, the protective role of TCA cycle metabolites under oxidative stress conditions has been also discussed. For example, the role of malate in the induction of protective antioxidant defense was demonstrated in green algae *Scenedesmus obliquus* and banana fruits [97,98], and citrate presence in the exudate of aluminum-tolerant *Indica* rice cultivars was shown to contribute to the tolerance of aluminum-induced oxidative stress [99].

### 3.3. Amino Acids

Amino acids are the building blocks of proteins and substrates for numerous secondary metabolites’ biosynthesis. Amino acid profile has been shown to be significantly affected by oxidative stress in plants (Figure 1) [20,24,25,28,29]. An increase in the content of individual amino acids during stress can result from the activation of biosynthetic pathways and from enhanced protein degradation [14,25,100]. The high contribution of protein degradation to the pool of free amino acids during oxidative stress has been confirmed in the analysis of labeled isotopes redistribution, revealing the suppression of the activity of pathways associated with amino acids metabolism [25,28]. Analysis of the available microarray dataset and amino acid profiles showed that under various abiotic stress conditions, abundant amino acids such as proline, arginine, asparagine, glutamine, and gamma-aminobutyric acid (GABA) are synthesized during abiotic stresses to be used by the plant as compatible osmolytes, precursors for secondary metabolites, or storage forms of organic nitrogen, while low abundance amino acids are not synthesized, but accumulate due to the increased protein degradation under stress-induced carbohydrate starvation [100].

The shikimate pathway, derived from phosphoenolpyruvate, the precursor of pyruvate, leads to the formation of aromatic amino acids and various secondary metabolites possessing antioxidant properties, such as lignin, flavonoids, alkaloids, and phytoalexins. Phenolic compounds containing one (phenolic acids) or more (polyphenols) aromatic rings, such as caffeic acid, chlorogenic acid, and ferulic acid, are effective antioxidants and free radical scavengers, and accumulation of these compounds is one of the most effective strategies used by plants to avoid oxidative damage [101,102,103]. Aromatic amino acids are used for the production of pigments, hormones, various polyphenols, and cell wall components [104]. Thus, these amino acids are directly and indirectly associated with antioxidant defense in plants. It has been demonstrated that phenylalanine and tyrosine accumulate in *Arabidopsis* roots under oxidative stress conditions [25], and the activation of the Shikimate pathway has been observed in *Scrophularia striata* cell culture under jasmonate-induced oxidative stress [105].

An increase in the branched-chain amino acids isoleucine and valine has also been observed in the roots of menadione-treated plants [25]. These amino acids are critical for normal plant growth, while also serving as substrates for protective secondary metabolites, such as cyanogenic glycosides, glucosinolates, and acyl-sugars [106].

Sulfur-containing amino acids, free and in proteins, together with other sulfur-containing metabolites (especially glutathione), represent an important part of the antioxidant system in animal and plant cells [107,108,109]. Methionine and cysteine are the major end products of sulfate assimilation comprising up to 90% of the total sulfur in most plants [110]. Based on the analysis of a ^13^C-redistribution in roots of plants supplied with ^13^C isotope labeled glucose, a significant decrease in methionine occurred under oxidative stress conditions, while the level of O-acetylserine, the precursor of cysteine, was increased significantly [24,25,28]. This suggests a shift in the balance in sulfur metabolism under stress conditions, which is most likely directed toward the production of cysteine, a substrate for glutathione biosynthesis. It was shown that the synthesis of glutathione itself, as well its key precursor cysteine, can be modulated by the ratio between reduced and oxidized glutathione [111,112]. Moreover, exogenously applied cysteine alleviates the oxidative stress induced by cobalt in *Ocimum basilicum* L. [113].

The level of alanine usually correlates to the intensity of starch catabolism. In the roots of menadione-treated *Arabidopsis* plants, alanine level was increased [25], although it did not show any significant response to ^13^C-redistribution in labeled glucose feeding experiments [28]. It was stated that the maintenance of high alanine levels under oxidative stress conditions correlates with plant tolerance to this stress [17].

Glutamate and aspartate derived from the TCA cycle are important substrates for the synthesis of several amino acids and donors of the amino group. The levels of aspartate derived from oxaloacetate and glutamate derived from 2-oxoglutarate decreased drastically in *Arabidopsis* roots under oxidative stress conditions induced by menadione treatment [25,28]. Importantly, in contrast to other metabolites, the levels of these amino acids did not return to the initial level, even during plant recovery from stress. At the same time, the downstream products asparagine and threonine showed an increase [25]. Glutamate and aspartate levels were also significantly decreased in menadione-treated rice suspension cells, despite an increase in most other amino acids, even glutamate and aspartate derivatives [14]. In *Arabidopsis*, oxidative stress induced by ultraviolet B (UV-B) treatment [114] moderately increased glutamate, and very strongly increased glutamine [115]. It seems that the ability to maintain the level of aspartate and glutamate under oxidative stress conditions also correlates with plant tolerance [17]. The increase in the levels of derivatives of glutamate and aspartate has been observed in several studies [14,25,29,115]. In wheat, TCA cycle-derived aspartic acid, asparagine, and GABA accumulated under oxidative stress conditions [29]. GABA was also strongly increased under oxidative stress in *Arabidopsis* roots [28]. The GABA often acts as a signal molecule in the regulation of stress responses, and antioxidant properties of GABA have been suggested [116].

In *Scrophularia striata* suspension cells the levels of asparagine and also histidine increased under oxidative stress induced by methyl jasmonate application [105]. UV-B treatment also significantly increased the level of lysine and histidine in *Arabidopsis* [115].

Proline, a general stress response metabolite associated with chloroplasts and photosynthesis [8], was reported to decrease in *Arabidopsis* cell culture treated with H_2_O_2_ [24], but increased in roots experiencing oxidative stress [25,28]. Oxidative stress resulting from aluminum toxicity was accompanied by a significant increase in proline in aluminum-tolerant rice cultivars [99]. The accumulation of proline under oxidative stress conditions and its role in the maintenance of redox balance have been demonstrated, although the ROS-scavenging ability of proline is still debated [117,118]. The level of glycine, another abundant stress response metabolite, was also decreased under oxidative stress conditions in *Arabidopsis* cell culture treated with H_2_O_2_ [24].

Citrulline is a nonessential amino acid that has been reported to be an efficient hydroxyl radical scavenger and a strong antioxidant [119,120]. Citrulline accumulation correlated with the tolerance to salt and drought stress [121,122]. Ornithine, the precursor of citrulline, was shown to increase transiently under oxidative stress [28].

The data on the major central metabolites involved in oxidative stress response, possessing antioxidant properties, or whose levels are affected by oxidative stress are summarized in Table 1. Thus, in this review, we have collected and analyzed the available data on oxidative stress-associated changes of the central metabolism in model plants and agricultural species. A thorough examination of the available data reveals common trends in the redistribution of metabolic fluxes during the plant adaptation to oxidative stress, targeted to the enhancement of plant performance under unfavorable environmental conditions. Adaptive metabolic changes are aimed at three major targets: (1) prevention of ROS accumulation, (2) maintenance of the biosynthesis of indispensable metabolites, and (3) production of protective compounds. The presented analysis provides the theoretical basis for the selection/generation of plants with improved tolerance to oxidative stress and the development of metabolic markers applicable in research and routine agricultural practice.

## 4. Conclusions

Plants constantly experience oxidative stress, and the ability to cope with this challenge largely determines plant survival, physiological health, and productivity. Chloroplasts are the major sites of ROS generation in plant cells [3]. The stress responses and redox disturbance within chloroplasts were shown to be stronger than in other compartments [8]. At the same time, the greatest part of the metabolic processes, including the biosynthesis of amino acids, fatty acids, lipids, vitamins, isoprenoids, most sulfur- and nitrate-containing compounds, occur in chloroplasts [123,124,125]. Undoubtedly, it is the processes occurring in chloroplasts that determine the strategy and the success of plant adaptation to stress conditions. This is why questions related to the formation of ROS in chloroplasts, as well as how it affects photosynthesis and various metabolic processes in plastids, and ways to improve the stability of chloroplast-localized molecular complexes, will remain the focal point of plant biology research.

## Figures and Tables

**Table 1:** A summary of the data on central metabolites associated with oxidative stress.

Metabolite	Studied System	Conditions	Description/Observed Effect	Ref.
sucrose	in vitro	-	free radicals-scavenging	[56,59]
	*Arabidopsis* cell culture	H_2_O_2_ treatment	accumulation upon H_2_O_2_ treatment	[24]
	*Phoebe bournei*, *Phoebe zhennan*	ozone-induced oxidative stress	accumulation upon ozone treatment (~100 nL/L)	[63]
	*Arabidopsis thaliana*	waterlogging-induced oxidative stress	stress-induced accumulation in leaves	[21]
glucose	in vitro	-	free radicals-scavenging	[56]
	*Solanum tuberosum*	hypothermia-induced oxidative stress	improvement in stress tolerance	[64]
	*Arabidopsis thaliana,* leaf protoplasts	H_2_O_2_ treatment	mitigation of oxidative stress	[65]
	*Nicotiana benthamiana*	H_2_O_2_- and α-picolinic acid–induced cell death	signaling in oxidativestress–induced cell death	[66]
	*Arabidopsis thaliana*	waterlogging-induced oxidative stress	stress-induced accumulation in leaves	[21]
fructose	fish oil-in-water emulsion	accelerated oxidation	ROS-scavenging, metal chelating	[52]
	in vitro	-	free radicals-scavenging	[56]
	*Arabidopsis* cell culture	H_2_O_2_ treatment	increase in abundance	[24]
	*Solanum tuberosum*	hypothermia-induced oxidative stress	improvement in stress tolerance	[64]
	*Arabidopsis thaliana,* leaf protoplasts	H_2_O_2_ treatment	mitigation of oxidative stress	[65]
RFO	*Arabidopsis thaliana*	waterlogging-induced oxidative stress	stress-induced accumulation in leaf tissue	[21]
	in vitro	-	free radicals-scavenging	[56]
	*Arabidopsis thaliana*	methyl viologen (MV) treatment	increased stress tolerance, ROS scavenging	[68]
	Mutant *Arabidopsis thaliana*	UV-B-induced oxidative stress	mediates oxidative stress response, regulates PCD	[80,81,82]
*myo*-inositol	in vitro	-	free radicals -scavenging	[60]
	*Arabidopsis thaliana*	waterlogging-induced oxidative stress	stress-induced accumulation in leaf tissue	[21]
	*Malus hupehensis*	growth under normal conditions	regulates ROS-induced programmed cell death	[79]
trehalose	*Triticum aestivum* L.	drought-induced oxidative stress	Exogenous application mitigates oxidative stress	[87]
	*Chenopodium quinoa*	drought-induced oxidative stress	Exogenous application mitigates oxidative stress	[88]
	*Lycopersicon esculentum*	methyl viologen treatment	Endogenous accumulation improves stress tolerance	[89]
fructans	in vitro	-	free radicals-scavenging	[54,56]
	intestinal lumen	-	antioxidant properties	[90]
	*Lolium perenne*	Drought, exogenous nitric oxide	Accumulation in tissue, mitigation of stress	[91]
citrate	*Oryza sativa* suspension cells	menadione treatment	decrease in abundance	[14]
	*Triticum aestivum* L.	chlorinated organophosphate esters-induced oxidative stress	increase in abundance	[29]
	heterotrophic *Arabidopsis* cells	H_2_O_2_ treatment	decrease in abundance	[24]
	*Arabidopsis thaliana* roots	menadione treatment	transient decrease followed by increase	[25,28]
	*Arabidopsis thaliana*	methyl viologen treatment	increase in abundance	[17]
	*Oryza sativa*	Al-induced oxidative stress	presence in exudate, improves stress-tolerance	[99]
aconitate	*Oryza sativa* suspension cells	menadione treatment	decrease in abundance	[14]
	*Triticum aestivum* L.	chlorinated organophosphate esters-induced oxidative stress	increase in abundance	[29]
	*Arabidopsis thaliana*	methyl viologen treatment	increase in abundance	[17]
isocitrate	*Oryza sativa* suspension cells	menadione treatment	decrease in abundance	[14]
	heterotrophic *Arabidopsis* cells	menadione treatment	decrease in abundance and/or formation	[9]
2-oxo-glutarate	*Oryza sativa* suspension cells	menadione treatment	decrease in abundance	[14]
	*Arabidopsis thaliana*	methyl viologen treatment	decrease in abundance	[17]
	*Arabidopsis thaliana* roots	menadione treatment	level changes dynamically	[28]
succinate	*Oryza sativa* suspension cells	menadione treatment	increase in abundance	[14]
	heterotrophic *Arabidopsis* cells	menadione treatment	decrease in abundance and/or formation	[9]
	heterotrophic *Arabidopsis* cells	H_2_O_2_ treatment	increase in abundance	[24]
	*Arabidopsis thaliana* roots	menadione treatment	decrease in abundance	[25,28]
	*Arabidopsis thaliana*	methyl viologen treatment	decrease in abundance	[17]
fumarate	*Oryza sativa* suspension cells	menadione treatment	decrease in abundance	[14]
	*Arabidopsis thaliana* roots	menadione treatment	decrease in abundance	[25,28]
	*Arabidopsis thaliana*	methyl viologen treatment	decrease in abundance	[17]
malate	*Oryza sativa* suspension cells	menadione treatment	decrease in abundance	[14]
	*Triticum aestivum* L.	chlorinated organophosphate esters-induced oxidative stress	increase in abundance	[29]
	heterotrophic *Arabidopsis* cells	menadione treatment	decrease in abundance and/or formation	[9]
	heterotrophic *Arabidopsis* cells	H_2_O_2_ treatment	decrease in abundance	[24]
	*Arabidopsis thaliana* roots	menadione treatment	decrease in abundance	[25,28]
	*Arabidopsis thaliana*	methyl viologen treatment	decrease in abundance	[17]
	algae *Scenedesmus obliquus*	C60 aggregates-induced stress	increase in abundance	[97]
	banana fruit	exogenously applied malate	improvement in stress-tolerance	[98]
Tyr	*Zea mays*	drought-induced oxidative stress	accumulation in xylem sap	[101]
	*Arabidopsis thaliana* roots	menadione treatment	increase in abundance	[25,28]
	*Oryza sativa* suspension cells	menadione treatment	increase in abundance	[14]
	*Scrophularia striata* cell culture	methyl jasmonate treatment	increase in abundance	[105]
Phe	*Zea mays*	drought-induced oxidative stress	accumulation in xylem sap	[101]
	*Arabidopsis thaliana* roots	menadione treatment	increase in abundance	[25]
	*Triticum aestivum* L.	chlorinated organophosphate esters-induced oxidative stress	increase in abundance	[29]
	*Oryza sativa* suspension cells	menadione treatment	increase in abundance	[14]
	*Scrophularia striata* cell culture	methyl jasmonate treatment	increase in abundance	[105]
Trp	*Zea mays*	drought	accumulation in xylem sap	[101]
	*Oryza sativa*	decabromodiphenyl ether treatment	improves stress-tolerance	[20]
	*Arabidopsis thaliana* roots	menadione treatment	increase in abundance	[28]
	*Oryza sativa* suspension cells	menadione treatment	increase in abundance	[14]
Lys	*Arabidopsis thaliana* roots	menadione treatment	increase in abundance	[25,28]
	*Oryza sativa* suspension cells	menadione treatment	increase in abundance	[14]
	*Arabidopsis thaliana*	UV-B-induced oxidative stress	increase in abundance	[115]
Val	*Oryza sativa*	decabromodiphenyl ether treatment	improves stress-tolerance	[20]
	*Arabidopsis thaliana* roots	menadione treatment	increase in abundance	[25,28]
	*Oryza sativa* suspension cells	menadione treatment	increase in abundance	[14]
Leu	*Zea mays*	drought-induced oxidative stress	accumulation in xylem sap	[101]
Ile	*Zea mays*	drought-induced oxidative stress	accumulation in xylem sap	[101]
	*Arabidopsis thaliana* roots	menadione treatment	increase in abundance	[25,28]
	*Oryza sativa* suspension cells	menadione treatment	increase in abundance	[14]
Asp	*Zea mays*	drought-induced oxidative stress	accumulation in xylem sap	[101]
	*Arabidopsis thaliana* roots	menadione treatment	decrease in abundance	[25,28]
	*Triticum aestivum*	chlorinated organophosphate esters-induced oxidative stress	increase in abundance	[29]
	*Oryza sativa* suspension cells	menadione treatment	decrease in abundance	[14]
	*Scrophularia striata* cell culture	methyl jasmonate treatment	increase in abundance	[105]
Asn	*Zea mays*	drought-induced oxidative stress	accumulation in xylem sap	[101]
	*Arabidopsis thaliana* roots	menadione treatment	increase in abundance	[25,28]
	*Triticum aestivum*	chlorinated organophosphate esters- induced oxidative stress	increase in abundance	[29]
Thr	*Zea mays*	drought-induced oxidative stress	accumulation in xylem sap	[101]
	heterotrophic *Arabidopsis* cells	H_2_O_2_ treatment	increase in abundance	[24]
	*Arabidopsis thaliana* roots	menadione treatment	increase in abundance	[25]
Met	heterotrophic *Arabidopsis* cells	H_2_O_2_ treatment	decrease in abundance	[24]
	*Arabidopsis thaliana* roots	menadione treatment	decrease in abundance	[25,28]
	*Oryza sativa* suspension cells	menadione treatment	decrease in abundance	[14]
Cys	*Zea mays*	drought-induced oxidative stress	decrease in xylem sap	[101]
	*Arabidopsis thaliana*, *cat2* mutant	catalase-deficiency	increase in abundance in leaves	[112]
	*Ocimum basilicum*	exogenously applied cysteine	alleviates oxidative stress	[113]
Glu	*Arabidopsis thaliana* roots	menadione treatment	decrease in abundance	[25,28]
	*Oryza sativa* suspension cells	menadione treatment	decrease in abundance	[14]
	*Arabidopsis thaliana*, *cat2* mutant	catalase-deficiency	increase in abundance in leaves	[112]
	*Arabidopsis thaliana*, *vtc1* mutant	UV-B-induced oxidative stress	increase in abundance	[114]
Gln	*Arabidopsis thaliana*	UV-B-induced oxidative stress	increase in abundance	[115]
Arg	*Oryza sativa* suspension cells	menadione treatment	increase in abundance	[14]
GABA	*Arabidopsis thaliana* roots	menadione treatment	increase in abundance	[28]
	*Triticum aestivum* L.	chlorinated organophosphate esters-induced oxidative stress	increase in abundance	[29]
	*Oryza sativa* suspension cells	menadione treatment	decrease in abundance	[14]
	*Arabidopsis thaliana*	elicitor-induced ROS burst	priming effect	[116]
Pro	*Zea mays*	drought-induced oxidative stress	accumulation in xylem sap	[101]
	heterotrophic *Arabidopsis* cells	H_2_O_2_ treatment	decrease in abundance	[24]
	*Arabidopsis thaliana* roots	menadione treatment	increase in abundance	[25,28]
	*Oryza sativa* suspension cells	menadione treatment	decrease in abundance	[14]
	*Ocimum basilicum*	treatment with cobalt nitrate	increase in shoots	[113]
	*Oryza sativa*	Al-induced oxidative stress	increase in abundance	[99]
Ornithine	*Arabidopsis thaliana* roots	menadione treatment	transient increase	[28]
Citrulline	in vitro	-	ROS-scavenging activity	[119]
Ala	*Arabidopsis thaliana* roots	menadione treatment	increase in abundance	[25,28]
	*Oryza sativa* suspension cells	menadione treatment	increase in abundance	[14]
	*Arabidopsis thaliana*, *rcd1* mutant	methyl viologen treatment	increased in stress-tolerant *rcd1* mutant	[17]
Gly	heterotrophic *Arabidopsis* cells	H_2_O_2_ treatment	decrease in abundance	[24]
	*Oryza sativa* suspension cells	menadione treatment	increase in abundance	[14]
	*Arabidopsis thaliana*, *cat2* mutant	catalase-deficiency	increase in abundance in leaves	[112]
His	*Oryza sativa* suspension cells	menadione treatment	increase in abundance	[14]
	*Scrophularia striata* cell culture	methyl jasmonate treatment	increase in abundance	[105]
	*Arabidopsis thaliana*	UV-B-induced oxidative stress	increase in abundance	[115]
Ser	*Zea mays*	drought-induced oxidative stress	accumulation in xylem sap	[101]
	heterotrophic *Arabidopsis* cells	H_2_O_2_ treatment	increase in abundance	[24]
	*Arabidopsis thaliana*, *cat2* mutant	catalase-deficiency	increase in abundance in leaves	[112]
*O*-acetyl-serine	*Arabidopsis thaliana*, *cat2* mutant	catalase-deficiency	increase in abundance in leaves	[112]
